# Diagnosis and Treatment of Hypermobile Cecum Mimicking Acute Appendicitis

**DOI:** 10.7759/cureus.86180

**Published:** 2025-06-16

**Authors:** Malak Maaz Hassan

**Affiliations:** 1 General Surgery, Hayatabad Medical Complex, Peshawar, PAK

**Keywords:** cecal volvulus, cecopexy, diagnosis of acute appendicitis, diagnostic laparoscopy, hypermobile cecum

## Abstract

A hypermobile cecum is a rare anomaly where incomplete fixation to the body wall causes increased mobility of the cecum and ascending colon. This condition can be asymptomatic or present with vague symptoms that can mimic other acute surgical conditions.

This is a case report of a 34-year-old female patient, who presented to the emergency department with clinical symptoms suggestive of acute appendicitis like right iliac fossa pain, vomiting and nausea. After an inconclusive ultrasound, a CT scan was performed, which revealed a hypermobile cecum with volvulus. An emergency laparoscopy was performed, during which both a successful cecopexy and appendectomy were carried out. The patient recovered well post-operatively and was discharged on the fourth post-operative day.

This case highlights the need for radiological imaging and the diagnostic difficulty caused by a hypermobile cecum, since it can resemble other common acute surgical situations. CT imaging is critical for precise diagnosis, and early surgical intervention is key to preventing complications such as perforation. Awareness of this condition is important for clinicians managing patients with unexplained right lower quadrant pain.

## Introduction

A hypermobile cecum is a rare developmental anomaly that occurs due to failure of attachment of the cecum and ascending colon to the posterior body wall [1]. Bowel development starts in the fifth week of gestation and involves three main stages: herniation outside the abdominal cavity, return to the abdomen, and fixation to the body wall [2,3]. Anomalies in rotation and fixation of the developing bowel may lead to failure of fixation of the cecum and ascending colon to the posterior abdominal wall, resulting in increased mobility of the cecum and the ascending colon. 

This condition can be asymptomatic but can present with vague symptoms such as recurrent right lower quadrant pain, abdominal distension, pseudo-obstruction, and, in some cases, volvulus, a serious condition requiring emergency surgery [4,5].

Owing to its atypical clinical presentation and rarity, it is usually confused with other acute surgical presentations. Radiological investigation plays an important role in diagnosis because of the non specificity of the clinical findings and laboratory tests for both mobile cecum and acute presentations. Diagnosis is often missed or delayed, posing a challenge to both clinicians and radiologists [1].

Simply fixation and suturing the cecum to the lateral peritoneum and interposition with a sponge have become outdated, and laparoscopic cecopexy is the treatment of choice now a days [4].

This case report highlights a patient with a symptomatic hypermobile cecum, emphasizing the importance of considering the differential diagnosis of this entity in recurrent or unexplained abdominal pain. A review of the diagnostic modalities and surgical management is also discussed.

## Case presentation

A 34-year-old female patient with a known history of gastroesophageal reflux disease (GERD) and depression presented to the emergency department with a 12-hour history of progressively worsening right lower quadrant abdominal pain. The pain was sharp, severe, and non-radiating, and was accompanied by nausea and one episode of non-bilious vomiting. She denied any changes in bowel habits, urinary symptoms, or recent travel. There was no history of fever, anorexia, or vaginal discharge.

On examination, she was tachycardic and afebrile, with stable vital signs. Abdominal examination revealed severe tenderness in the right lower quadrant with positive guarding and Rovsing’s sign. She underwent routine blood investigations, which showed an elevated white blood cell count. C-reactive rotein (CRP), electrolytes, and renal function were normal (Tables 1, 2).

**Table 1:** Complete blood count RDW: Red cell distribution width; RBC: Red blood cell

Test	Result	Normal Range
White Cell Count	9.2×10^9^/L	3.5-11×10^9^/L
Hemoglobin	13.7 g/dL	11.5-15 g/dL
Platelets	293×10^9^/L	150-400×10^9^/L
Red Cell Count	4.53×10^12^/L	3.8-4.8×10^12^/L
Hematocrit	0.386 L/L	0.37-0.47 L/L
Mean Cell Volume	85 fL	80-100 fL
Mean Cell Hemoglobin	30.2 pg	27-32 pg
RDW	12%	11-15%
Neutrophils	6×10^9^/L	2-8×10^9^/L
Lymphocytes	2.6×10^9^/L	1-4×10^9^/L
Monocytes	0.4×10^9^/L	0.2-1.0×10^9^/L
Eosinophils	0.1×10^9^/L	0.0-0.5×10^9^/L
Basophils	0.0×10^9^/L	0.0-0.2×10^9^/L
Nucleated RBCs	0.0×10^9^/L	0.0-0.01×10^9^/L

**Table 2:** Liver function tests, renal function tests, and serum profile GFR: Glomerular filtration rate; ALB: Albumin; ALT: Alanine aminotransferase; GGT: Gamma glutamyl transferase; CRP: C-reactive protein

Test	Result	Normal Range
Sodium	141 mmol/L	133-146 mmol/L
Potassium	4.6 mmol/L	3.5-5.3 mmol/L
Chloride	105 mmol/L	95-108 mmol/L
Total CO_2_	26 mmol/L	22-29 mmol/L
Urea	3.4 mmol/L	2.5-7.8 mmol/L
Creatinine (Enzymatic)	67 umol/L	45-84 umol/L
Estimated GFR	≥90 mL/min/1.73sq.m	
Total Protein	76 g/L	60-80 g/L
Calculated Globulin	33 g/L	25-40 g/L
ALB	43 g/L	35-50 g/L
Total Bilirubin	5 umol/L	0-21 umol/L
Alkaline Phosphate	65 IU/L	30-130 IU/L
GGT	22 IU/L	6-42 IU/L
ALT	19 IU/L	8-41 IU/L
Amylase	70 IU/L	28-100 IU/L
CRP	3.3 mg/L	

An abdominopelvic ultrasound was performed, which was inconclusive because of overlying bowel gas, and she was booked for a CT scan of the abdomen and pelvis to evaluate her symptoms. Given the typical symptoms of abdominal pain, rebound tenderness and Rovsing’s sign, a clinical diagnosis of acute appendicitis was established.

**Figure 1:**
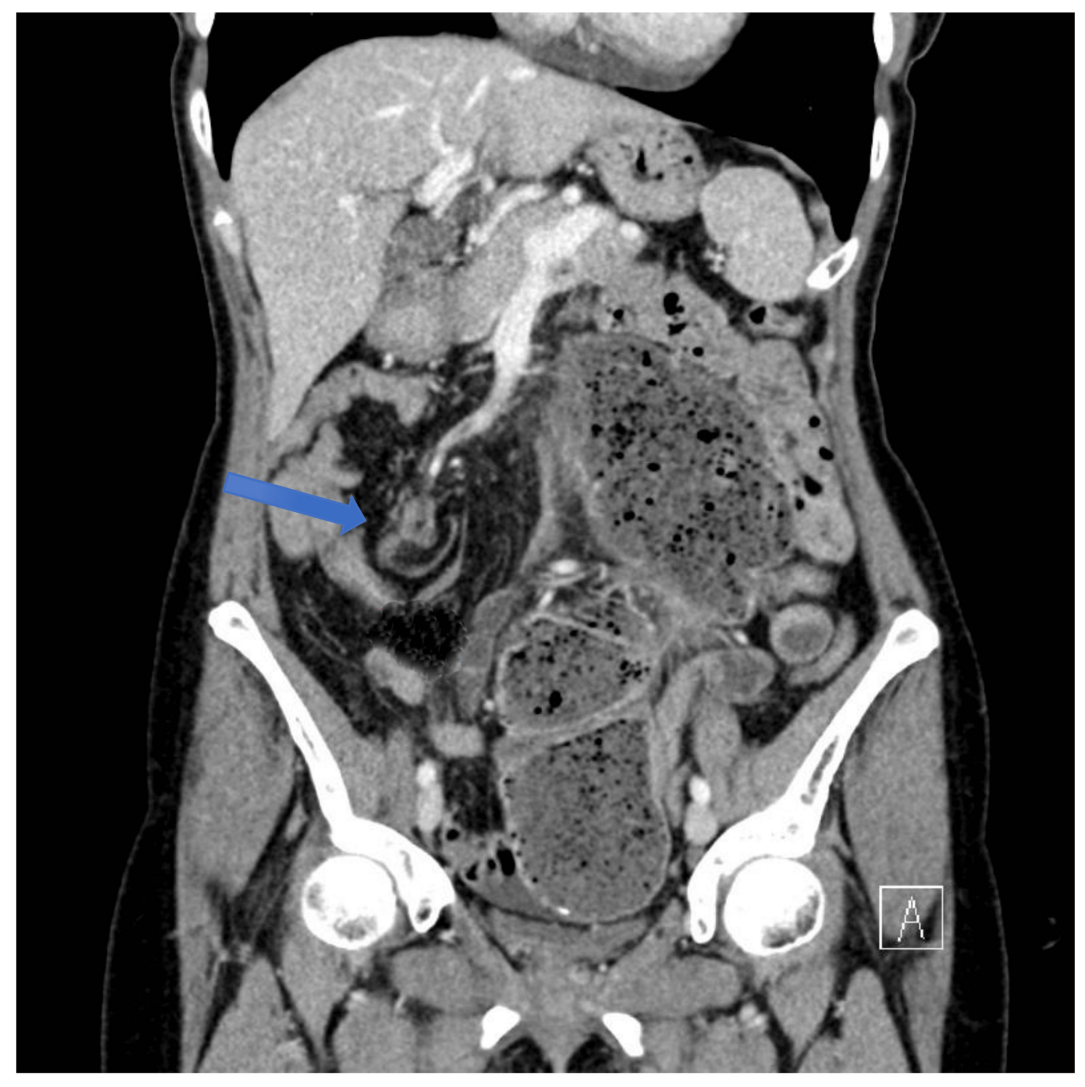
CT scan of abdomen and pelvis, showing cecal volvulus The blue arrow points to whirls, indicating twisting of cecal mesentery.

When the CT scan was done, it showed cecal volvulus, hypermobile caecum, with the caecal pole lying just to the left of midline at the level of the umbilicus and no evidence of acute appendicitis (Figure 1). She was immediately booked for an emergency laparoscopy.

Laparoscopy revealed distension of the cecum, ileum, and jejunal loop. The colon beyond the point of rotation was collapsed. The cecum was twisted along its mesentery and located in the umbilical region. A microbiological culture was taken from the abdominal fluid, which was reported as negative. After untwisting of the cecum, the bowel showed complete recovery; therefore, an appendectomy and cecopexy were carried out. The patient recovered well post-operatively and was observed in the surgical ward. The patient was discharged on the fourth post-operative day.

## Discussion

The hypermobile cecum remains asymptomatic; however patients may present with intermittent or chronic right lower quadrant abdominal pain, often mimicking appendicitis or other gastrointestinal disorders. Volvulus of the mobile cecum requires urgent surgical intervention. If not treated promptly, it can lead to serious complications such as bowel ischemia, gangrene, and perforation. Therefore, early and accurate diagnosis is crucial.

As clinical examination and laboratory tests are often non-specific, radiological investigations play a central role in diagnosis. Ultrasound is the first investigation that’s usually carried out because of its cost-effectiveness, easy availability, and the ability to exclude various surgical conditions like appendicitis or macroscopic lesions of the intestine. However, its findings are generally non-specific [6]. A CT scan is highly specific for the mobile cecum and reveals suspicion of cecum malposition [7].

Laparoscopy remains a valuable tool not only for diagnosis but also for therapeutic management. Surgical intervention depends on the patient's clinical presentation and bowel viability. Cecopexy is only suitable for volvulus if the bowel is viable, but in case of gangrenous and a grossly distended, thin-walled cecum, resection become mandatory [8]. Given the absence of ischemia in our patient, cecopexy was performed. Some surgeons advocate performing cecopexy to prevent future complications; however, there is limited consensus due to the rarity of the condition.

## Conclusions

A hypermobile cecum is a rare condition that can mimic other acute surgical pathologies, making diagnosis challenging. It should be considered in the differential diagnosis of right lower quadrant abdominal pain. Clinical identification of a mobile cecum is often difficult; therefore, a CT scan is the preferred imaging modality for accurate diagnosis. The recommended treatment is cecopexy using a peritoneal flap along with an appendectomy, which helps resolve cecal volvulus and prevent recurrence.
